# Evaluation of a protocol to detect malnutrition and provide nutritional care for cancer patients undergoing chemotherapy

**DOI:** 10.1038/s41598-020-78246-w

**Published:** 2020-12-03

**Authors:** Elena Álvaro Sanz, Jimena Abilés, Marga Garrido Siles, Francisco Rivas Ruíz, Begoña Tortajada Goitia, Antonio Rueda Domínguez

**Affiliations:** 1 Pharmacy and Nutrition Service, Hospital Costa del Sol, Marbella, Málaga Spain; 2 Pharmacy Service, Hospital Universitario Virgen de La Victoria, A7, km. 187, Área de Farmacia, Campus de Teatinos, S/N, 29010 Málaga, Spain; 3 Research Department, Hospital Costa del Sol, Marbella, Spain; 4 UGCI Oncología Médica, Hospitales Universitarios Regional Y Virgen de La Victoria, IBIMA, Málaga, Spain; 5 Málaga, Spain

**Keywords:** Cancer, Signs and symptoms, Oncology

## Abstract

Patients with cancer frequently experience malnutrition, which is associated with higher rates of morbidity and mortality. Therefore, the implementation of strategies for its early detection and for intervention should improve the evolution of these patients. Our study aim is to design and implement a protocol for outpatients starting chemotherapy, by means of which any malnutrition can be identified and treated at an early stage. Before starting chemotherapy for patients with cancer, a complete assessment was made of their nutritional status, using the Nutriscore screening tool. When nutritional risk was detected, an interventional protocol was applied. Of 234 patients included in the study group, 84 (36%) required an individualised nutritional approach: 27 (32.1%) presented high nutritional risk, 12 had a Nutriscore result ≥ 5 and 45 experienced weight loss during chemotherapy. Among this population, the mean weight loss (with respect to normal weight) on inclusion in the study was − 3.6% ± 8.2. By the end of the chemotherapy, the mean weight gain was 0% ± 7.3 (*p* < 0.001) and 71.0% of the patients had experienced weight gain or maintenance, with respect to the initial weight. More than a third of cancer patients who start chemotherapy are candidates for early nutritional intervention. This finding highlights the importance of early identification of patients at risk in order to improve the efficacy of nutritional interventions, regardless of the stage of the disease.

## Introduction

Cancer is highly prevalent and a major cause of morbidity and mortality worldwide, impacting severely on health systems and on patients’ quality of life (QoL)^1^.

In recent years, survival rates have improved, thanks to early diagnosis and more effective treatments. In this scenario of long-term survival, health care should not focus exclusively on the disease, but address all aspects of the patient’s condition. In other words, oncological care is evolving to become a multidisciplinary model incorporating a wide range of services and concerns^2^.

Within this broader approach, weight loss (WL) and other signs of deteriorating nutritional status require special attention, due to their impact and prevalence, and so action protocols should be established to promote comprehensive nutritional care^2^.

The latest recommendations on nutritional care for cancer patients emphasise the importance of detection and prompt action for persons at nutritional risk, to prevent the onset of malnutrition and to minimise its devastating effect on the patient’s clinical condition^3^.

Early action is especially important due to the evolutionary nature of oncological cachexia and the probability of its becoming refractory and irreversible^4^. In this respect, studies have shown that early nutritional intervention can reduce the catabolic impact of cachexia, resulting in clinical improvement and enhancing survival rates for cancer patients at high nutritional risk, such as those with oesophageal or gastric tumours^5,6^.

In 2016, the Pharmacy and Nutrition Service at our hospital designed and implemented a nutritional care model for oncology patients^7^ to enable the early detection of nutritional risk, to facilitate periodic assessment and nutritional monitoring, and to provide nutritional intervention at an early stage, prior to the appearance of refractory cachexia.

The aims of the present study are to determine the effectiveness of this nutritional care model for cancer patients, in terms of weight gain or maintenance by the end of chemotherapy, and to promote the early detection of vulnerable patients.

## Methods

### Study population

Adult patients (18 years or older) with a de novo diagnosis of solid tumour, regardless of stage, who started chemotherapy at least 15 months previously, were included in the study. Those who, for cultural or cognitive reasons, had difficulty understanding the study aims were excluded. All patients who met the inclusion and exclusion criteria were included consecutively.

The study protocol was carried out in accordance with the provisions of the Declaration of Helsinki and was approved by the local Clinical Research Ethics Committee. All patients included in the study gave their written informed consent to participate.

### Study design

In this prospective study, the nutritional care model for cancer patients differentiates two groups of patients according to the location of the tumour and its impact on nutritional status: group 1 includes patients with high-risk tumours (cancers of the head and neck and of the upper digestive tract, oesophagus, stomach, pancreas or bile duct); group 2 contains patients with low nutritional risk tumours and all other malignancies.

In the course of the study, the group 1 patients were referred directly for nutrition consultation, either after presentation of their data to the corresponding oncology committee or during the admission in which they were diagnosed with the tumour pathology. The “Individualised nutritional care programme” was then launched, involving nutritional assessment, intervention and vigilant follow-up. This process begins with a complete nutritional assessment of food intake and symptoms that could affect nutritional status, following international guidelines of The European Society for Clinical Nutrition and Metabolism (ESPEN) on nutritional support for cancer patients^3^. The type and degree of malnutrition observed determines the nutritional intervention stipulated, ranging from nutritional advice to specialised nutritional support, as recommended in the ESPEN guidelines^3^.

The group 2 patients received a nutritional screening at their first consultation with the oncologist or at the day-patient hospital pharmacy consultation, before starting chemotherapy. Patients whose screening results were positive, showing them to be at risk of malnutrition, were assigned a degree of malnutrition, according to the patient-generated subjective global assessment (PG-SGA), a validated method that produces the following classifications: (A) Normally nourished; (B) At nutritional risk or presenting moderate malnutrition; (C) Presenting severe malnutrition^8^. Patients classed as B or C were referred to the nutrition consultation and the “Individualised nutritional care programme” was initiated.

**Figure 1:**
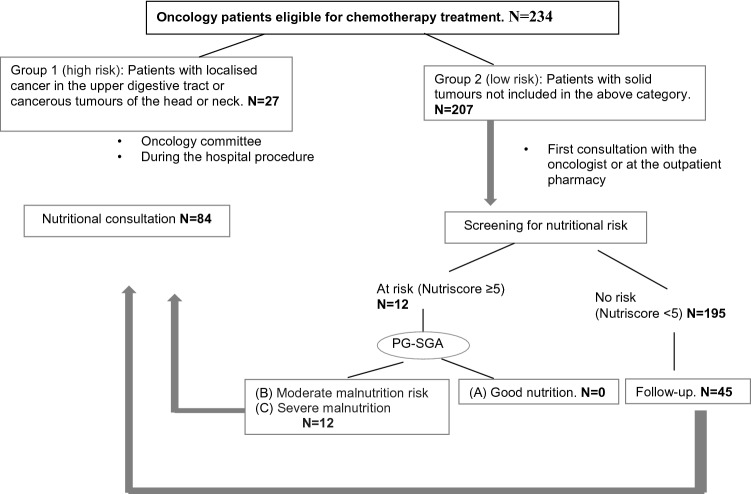
Nutritional care model.

Group 2 patients whose screening results were negative and who, therefore, were not considered to be at nutritional risk, received periodic re-evaluations, with continuous monitoring in each treatment cycle or during the perioperative period. All the patients included in this study were given nutritional recommendations on request, whether or not nutritional risk was observed. The procedure shown in Fig. 1 was followed and the steps followed are detailed in the Table 1.

**Table 1:** Nutritional care model for cancer patients.

Step 1	Tumour Committee: After consideration of the cases by the Committee, the patients are classified according to the type of tumour presented
Step 2	Patients with tumours of the head and neck, upper digestive tract, pancreas or bile ducts are classified as high risk (Group 1)
	Patients with other tumours (colon, lung, breast, gynaecological, etc.) are classified as low risk (Group 2)
Step 3	Group 1 (high risk). Rapid nutritional approach: within 3 days of presentation to the Tumour Committee, a treatment plan is established and individualised follow-up prepared
	Group 2 (low risk). Nutritional screening (Nutriscore) is performed at the start of cancer treatment
Step 4	Patients with Nutriscore ≥ 5. Nutritional status is assessed by PG-SGA as follows: Good nutritional status: weight control during each cycle of chemotherapy Risk of moderate or severe malnutrition: referral to nutrition clinic
	Patients with Nutriscore < 5. Reassessment during each chemotherapy cycle

*PG-SGA* patient-generated subjective global assessment.

A vigilant, individualised monitoring plan was then established, with periodic reviews to assess the patient’s treatment adherence, tolerance and efficacy. According to the treatment protocol, the periodicity of visits to the nutrition clinic was determined according to the tumour location. In most cases, these visits took place on day 1 of each chemotherapy cycle.

Patients at nutritional risk were identified using Nutriscore, a nutritional screening test for outpatients with cancer, which takes into account involuntary weight loss in the previous three months, decreased appetite, tumour location and oncology treatment. Patients were considered at risk when the Nutriscore result was 5 points or more (on a scale with a maximum score of 9 points). Nutriscore is a screening method that has been validated for use in the Spanish population by reference to the Patient-Generated Subjective Global Assessment (PG-SGA) instrument and the Malnutrition Score Tool (MST), with a sensitivity of 97.3% and a specificity of 95.9%^9^.

Each patient’s body weight was recorded at different times during the nutritional care, and was defined as follows:

- Normal weight (NW): Weight during the last three months, as reported by the patient.
- Initial weight (IW): Weight when the nutritional care model was first applied. This value is expressed as the percentage of WL (%WL) on first application of the nutritional care model.
- Chemotherapy start weight (CSW): Weight at the start of chemotherapy.
- Chemotherapy end weight (CEW): Weight on concluding chemotherapy.

The effectiveness of the nutritional care protocol was evaluated by reference to the percentage of weight gained (%WG) or maintained (%WM) at the end of the treatment, quantified as the difference between the patient’s weight on concluding chemotherapy and the initial weight recorded.

A WL of 2% or more was considered significant. Smaller losses were not taken into consideration, since they might reflect intra and interpersonal variability when the weight was recorded (for example, shortly after eating or fasting, or differences in the weight of clothing).

The nutritional care protocol was considered to have been applied at an early stage when the patient was first attended in this respect before starting chemotherapy or during the first seven days thereafter.

### Statistical analysis

Descriptive analysis was performed using measures of central tendency, dispersion and position for the quantitative variables and of frequency distribution for the qualitative ones. Differences between two measurements were evaluated by the Wilcoxon rank test or the Mann–Whitney U test for independent samples referring to quantitative variables. Average differences between two groups were evaluated by the chi-square test. In all cases, statistical significance was assumed at *p* < 0.05. An annual population of about 300 patients at our hospital are candidates to participate in the study. Following implementation of the nutrition protocol, 80% of these patients will either gain weight or maintain their original weight. To achieve a confidence level of 95% and a precision of 3%, a minimum sample size of 209 patients will be needed. This figure will be increased by 10% (to 230) to counteract losses to follow-up.

Finally, a multivariante logistic regression model was obtained, between the outcome variable (weight loss > 2% or weight gain/maintenance) at the end of chemotherapy treatment, with tumour type, age, sex and intention to treat as independent variables. The level of statistical significance was set at *p* < 0.05.

### Ethics approval

The study protocol was approved by the Medical Ethics Committee at the Hospital Costa del Sol Hospital.

### Consent to participate

All patients included in this study provided written informed consent.

### Consent for publication

Not applicable.

## Results

Of the initial 295 patients included in the study when the nutritional care model was initiated for cancer patients, 51 (17%) did not complete chemotherapy. In addition, there were ten losses to follow-up. Thus, 234 patients remained in the final analysis.

The nutrition consultation showed that 84 patients required individualised nutritional care: 27 (32.1%) belonged to group 1 (high-risk tumours), and another 12 had Nutriscore ≥ 5 at the start of treatment (the 27 patients with high-risk tumours also had Nutriscore ≥ 5), and 45 patients, although not at nutritional risk at the start of treatment, had recorded WL during one or more of the periodic re-evaluations, on day 1 of each cycle of chemotherapy. On average, each patient attended the nutrition consultation five times. The mean follow-up period was 6 months. 68% of the patients required enteral nutritional support, the most widely used enteral nutrition formulas being hypercaloric/high protein with and without fiber and diet formulas.

The clinical characteristics recorded at the start of chemotherapy, for all patients and also for those who required assessment, nutritional intervention and vigilant follow-up are shown in Table 2.

**Table 2:** Patients’ characteristics.

	All patients	Patients needing assessment, nutritional intervention and vigilant follow-up
	N (%)	(%)
**Overall**	234	84
**Sex**		
Male	136 (58.1)	33 (39.3)
Female	98 (41.9)	51 (60.7)
**Age (years), mean ± SD**	59 ± 11	60 ± 11
**Location of primary tumour**		
Head-neck	5 (2.1)	5 (6.0)
Colon-rectum	37 (15.8)	26 (31.0)
Oesophagus-stomach	13 (5.6)	13 (15.5)
Gynaecological	32 (13.7)	10 (11.9)
Breast	72 (30.8)	5 (6.0)
Pancreas-bile ducts	9 (3.8)	9 (10.7)
Lung	45 (19.2)	9 (10.7)
Urothelial	12 (5.1)	3 (3.6)
Other	9 (3.8)	4 (4.8)
**Treatment intention**		
Curative/radical	154 (65.8)	45 (52.4)
Palliative	80 (34.2)	39 (46.4)
**BMI (mean ± SD)**	26.6 ± 4.8	24.5 ± 4.0
**Initial % weight loss (NW-CSW) (mean ± SD)**	3.9 ± 7.3	10.1 ± 7.4
**Nutritional risk (Nutriscore)**		
≥ 5	39 (16.7)	39 (46.4)
< 5	195 (83.3)	4 (53.6)

*BMI* body mass index, *NW* normal weight, *CSW* chemotherapy start weight.

### Patients in group 1 and patients in group 2 with nutriscore ≥ 5 and PG-SGA B/C (n = 39)

**Figure 2:**
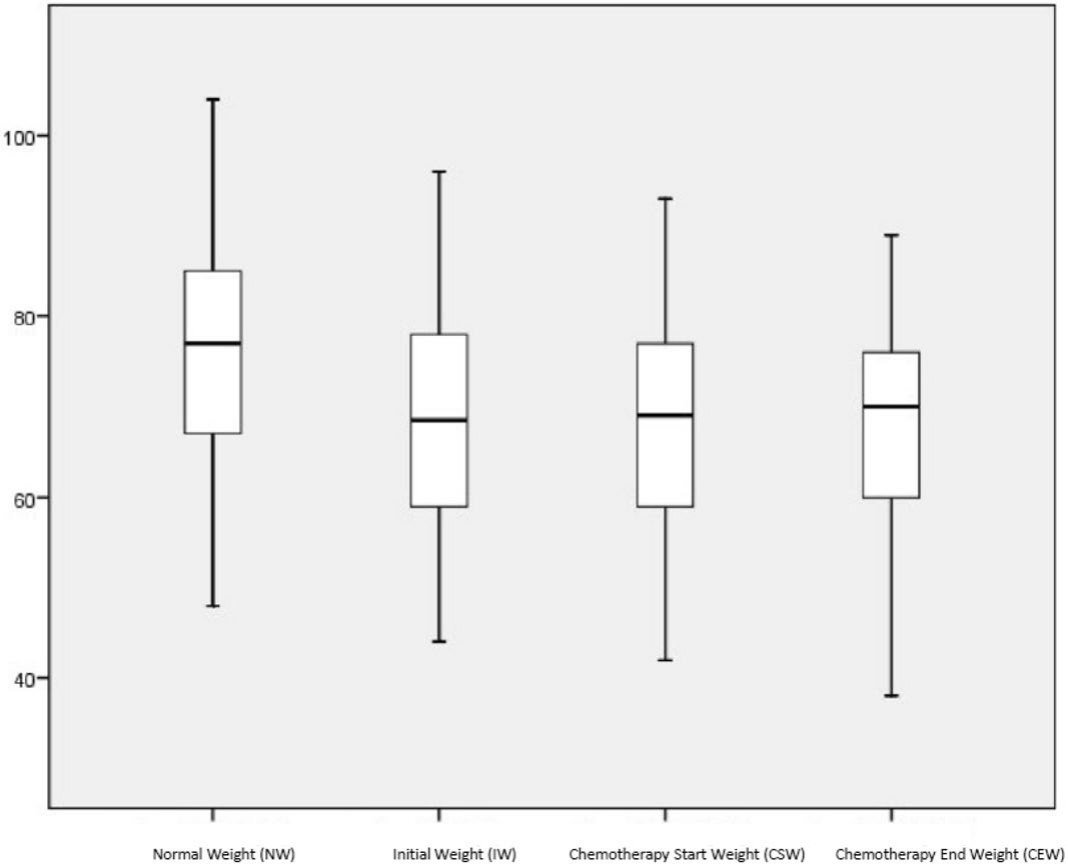
Evolution of body weight in patients in group 1 and 2(Nutriscore ≥ 5 and PG-SGA B/C). *NW* normal weigth, *IW* initial weigth, *CSW* chemotherapy start weigth, *CEW* chemotherapy end weigth.

On their first visit to the nutrition consultation, these patients presented a median %WL of 9.2% (IQR = 10.8) (with respect to normal weight). After establishing an individualised nutritional care plan, the median %WL (with respect to initial weight) was 0% (IQR = 1.6) at the start of chemotherapy. By the end of the chemotherapy programme, 58% of the patients presented weight gains or had maintained their previous weight (with respect to the initial weight). Figure 2 shows the evolution of these weights.

The median time elapsed from the first nutrition consultation and the start of the “Individualised nutritional care programme” until the start of chemotherapy was 26 days (IQR = 54). The median time elapsed from disease diagnosis until the first nutrition consultation was 9 days (IQR = 9).

### Group 2 patients not presenting nutritional risk at the start of chemotherapy, but subsequently with nutriscore ≥ 5 and PG-SGA B/C (n = 45)

Of the patients not assigned to group 1 (high-risk tumours) and who presented no deterioration in nutritional status at the start of treatment, 19% required individualised nutritional care after the post-chemotherapy re-evaluation detected WL and PG-SGA reflected the risk or presence of malnutrition.

**Figure 3:**
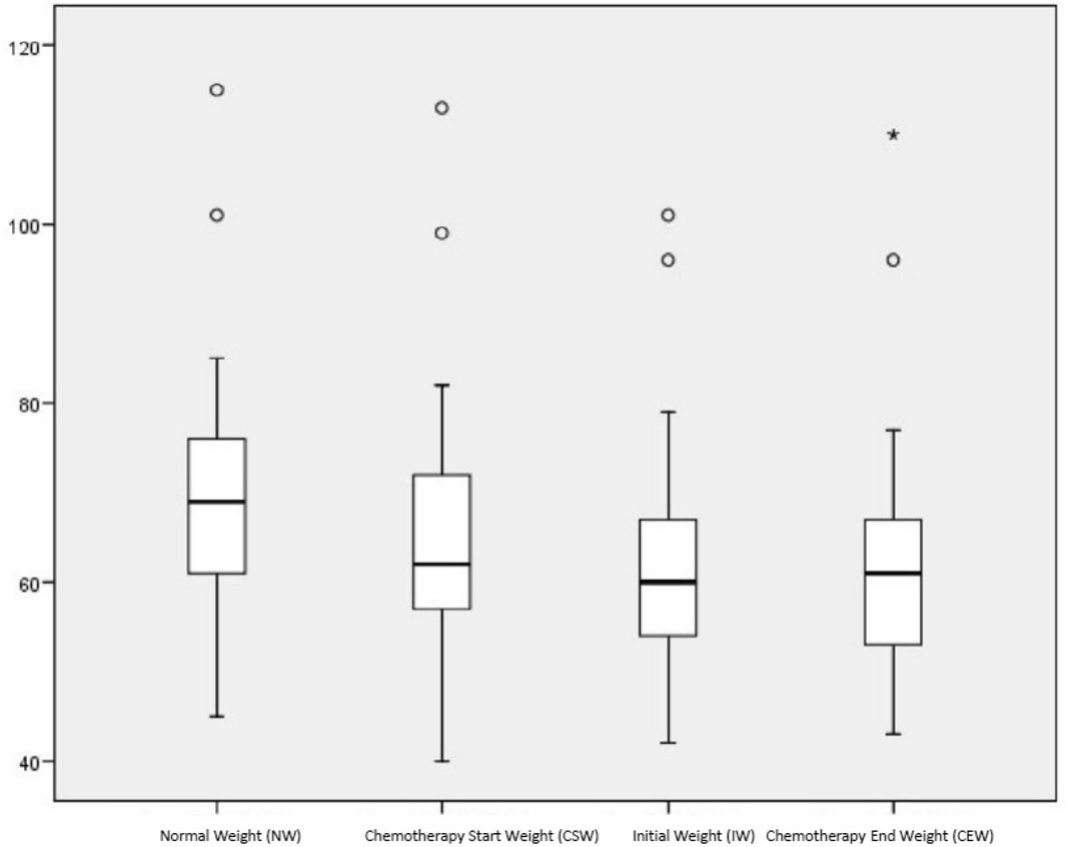
Evolution of weights in group 2 patients with Nutriscore ≥ 5 and PG-SGA B/C after starting chemotherapy. *NW* normal weigth, *CSW* chemotherapy start weigth, *IW* initial weigth, *CEW* chemotherapy end weigth.

These patients presented a median %WL of 6.1 (IQR = 11.6) at the start of chemotherapy (with respect to normal weight). During treatment, the %WL was 12.2 (IQR = 11.4) and the patients were referred to nutrition consultation. Figure 3 shows the evolution of the weights recorded for these patients. Of the 45 in follow up with score < 5, on concluding chemotherapy, 54% presented weight gains or maintained their previous weight.

Although on average the nutrition consultation took place 43 days (IQR = 85) after the start of chemotherapy, the time elapsed did not influence the effectiveness of the “Individualised nutritional care programme”. Although the nutritional programme was not applied at such an early stage to this group of patients, their rate of weight loss nevertheless slowed down considerably. With the nutritional approach, the degree of weight loss had fallen from 12.2 to 0.68% by the end of the treatment, with respect to the usual weight at the beginning of the protocol. These differences are statistically significant (*p* < 0.05).

Our analysis of the changes in the patients’ body weight showed that 71% of the sample gained weight or maintained their original weight by the end of the chemotherapy sessions (with respect to the initial weight). Moreover, about 80% of the patients with cancer of the head and neck, or gynaecological, breast or bladder cancer maintained or gained weight. The patients with oesophageal-gastric cancer or pancreatic cancer did not achieve weight gain, but in 46.2% and 44.4% of these cases, respectively, there was no further WL during treatment. Moreover, the amount of WL had decreased by the end of the treatment, with respect to the initial weight, although the difference was not statistically significant.

In the overall study sample, the mean %WL on inclusion in the “Individualised nutritional care programme” (with respect to normal weight) was − 3.6% ± 8.2. At the end of chemotherapy, the average weight gain was 0% ± 7.3, and the differences between the two periods were statistically significant (*p* < 0.001). At this time point, 71.0% of the study sample had experienced weight gain or had maintained their initial weight (Table 3).

**Table 3:** Percentage of weight loss at the start of treatment and of weight gain/maintenance by the end of treatment, according to tumour location.

Location (n)	% weight loss at the start of the protocol, with respect to normal weight (median, IQR)	% weight gained or maintained at the end of treatment (compared to start of protocol) (median, IQR)	Statistical significance (start of protocol vs. end of chemotherapy) *p* < 0.005
Head-neck (5)	− 16.3 (18.1)	1.7 (15.6)	0.080
Oesophagus-stomach (13)	− 7.8 (11.5)	− 2.9 (10.6)	0.101
Pancreas-bile ducts (9)	− 7.9 (14.5)	− 5.6 (12.5)	0.678
Colorectal (37)	− 6.1 (9.7)	0.0 (6.7)	0.002
Gynaecological (32)	− 5.2 (2.2)	1.7 (3.9)	0.003
Lungs (45)	− 0.6 (15.5)	0.0 (7.3)	0.044
Other (9)	− 3.8 (7)	0.0 (16.8)	0.028
Breast (72)	0.0 (5.8)	0.0 (4.8)	0.226
Bladder (12)	− 2.0 (5.8)	1.0 (4.1)	0.308
Total sample (234)	− 3.6% ± 8.2 (mean ± SD)	0% ± 7.3 (mean ± SD)	< 0.001

A multivariate logistic regression model was generated, revealing that none of the variables considered (tumour type, age, sex and intention) were associated with the outcome variable.

## Discussion

Although the considerable prevalence of malnutrition among cancer patients is well documented, as are the fact that it negatively impacts on the prognosis and that nutritional intervention improves patients’ survival and QoL^10^, many malnourished patients remain unidentified and hence are not treated appropriately.

The NUPAC study^11^, which to our knowledge is the only one carried out in Spain to determine the incidence of malnutrition among cancer patients, revealed that over 50% had moderate or severe degrees of malnutrition. However, the most alarming aspect reported of this study is the large number of cancer patients for whom no nutritional diagnosis is made. In this respect, Duran-Poveda et al. conducted a study using the Delphi method, involving 52 medical specialists who treated cancer patients, and found that fewer than 30% of these patients were screened to assess their risk of malnutrition^12^.

According to Kruizenga et al., the application of an early detection protocol, when the cancer is diagnosed, can improve the recognition of malnourished patients by 50–80%^13^.

In our study, application of the “Individualised nutritional care programme” to cancer patients enabled 100% of those with solid tumours and undergoing chemotherapy to be approached; of these, 36% were found to be at nutritional risk.

According to the ESPEN guidelines, the degree of WL is the most reliable indicator of nutritional deficit^3^. Forty years ago, studies reported that WL at diagnosis was common among cancer patients^14^ and was strongly related to poor outcomes at all stages of cancer^15^.

In 1980, as part of the ECOG study, De Wys et al. retrospectively evaluated WL in over 3000 cancer patients. Moderate to severe WL was observed in 40–80% of patients, according to the type of tumour. The frequency of WL was greater among patients with gastric or pancreatic neoplasia. This study was criticised for not including head and neck cancer patients in its study group^14^. In our own research, patients with head or neck cancer presented the highest %WL (%WL = 16.3), while gastric and pancreatic neoplasia were associated with higher rates of WL than other digestive tumours.

The standard practice of medical care must include nutritional assessment, which must be multidisciplinary and adapted to the special characteristics of each center. A multidisciplinary group of expert recommends using nutritional screening routinely, at diagnosis and throughout the disease course^16^. The main aim of this approach is to identify patients with malnutrition or at high risk of nutritional complications caused by the disease or by the treatment received. Our nutritional care model for cancer patients, an early approach is taken to monitor and treat patients at nutritional risk, based on applying the triple technique of assessment, nutritional intervention and vigilant follow-up, in many cases up to a month before starting chemotherapy.

Nutritional intervention is effective in reducing WL, alleviating the effects associated with malnutrition, reducing the incidence of hospital admissions and improving QoL. Therefore, it should be considered part of the standard treatment provided to cancer patients^10,17^. Nutritional intervention is more effective at earlier stages of the disease, before a state of refractory cachexia becomes established^5^.

Therefore, it is essential to monitor the patient’s weight throughout the treatment process. Our “Individualised nutritional care programme” for oncology patients detected 45 patients, who despite not obtaining a Nutriscore result of ≥ 5 points at diagnosis, presented WL during cancer treatment, which was detected by monitoring the patient’s weight on the first day of each cycle of chemotherapy. Accordingly, the hospital pharmacist, who sees patients in each of these cycles, plays a role of fundamental importance in the protocol.

A study was conducted in Spain with 997 patients, seeking to determine the prevalence and degree of malnutrition in cancer patients who had been referred to a nutrition consultation. The authors found that 57.5% of the patients were referred for consultation due to WL after starting chemotherapy, and that in 42.8% of these cases, the degree of WL was over 10%^18^.

Our results show that by identifying vulnerable patients (with high-risk tumours or identified as such by Nutriscore and PG-PGSA-B/C) and then applying assessment, nutritional intervention and vigilant follow-up, 70% of patients achieve weight gain or maintenance by the end of the treatment programme, which corroborates the effectiveness of our “Individualised nutritional care programme” for cancer patients.

Although for some types of cancer the differences were not statistically significant, our study shows that patients tend to recover from, or at least slow, their WL when the “Individualised nutritional care programme” is applied. Comparable data were reported by Bellestero-Pomar et al.^19^, who implemented a similar protocol for screening and nutritional intervention with hospitalised onco-haematological patients.

The fact that no weight gain was achieved (weight maintenance was the best result obtained) in the group of patients with high-risk tumours leads us to believe that perhaps a more intensive follow-up programme or a more aggressive nutritional intervention, such as home parenteral nutrition, should be applied.

### Limitations

This study is subject to certain limitations, which should be acknowledged. Firstly, due to ethical constraints, we were unable to establish a control group to properly evaluate the effectiveness of the protocol. In consequence, we can only compare our results with those published previously.

Furthermore, most published studies addressing issues of nutritional intervention refer to specific tumour lines, and most have been conducted focusing on tumour locations considered to produce a high risk of malnutrition. Our study encompasses the entire cancer population, which makes it more complex to compare our results with those published previously.

## Conclusions

The distinctive elements of our study are that it was carried out with outpatient patients and that the model proposed allows early nutritional care to be provided, regardless of tumour location, and so different levels of action may be taken according to the nutritional risk assessed. It is important to note that the early application of our protocol means that when chemotherapy is started the patient has an optimum nutritional status, which could favour treatment tolerance and facilitate weight recovery or maintenance during or after this process. We also show that an early, systematised and individualised approach can prevent or reduce the nutritional deterioration associated with chemotherapy. In fact, the model described has been incorporated into normal clinical practice at our hospital, enabling a comprehensive nutritional approach to be provided to all patients.

## Data Availability

The datasets used and/or analysed during the current study are available from the corresponding author on reasonable request.
